# Supplementary material for: Effects of stanniocalcin-1 overexpressing hepatocellular carcinoma cells on macrophage migration
Source: PLoS One. 2020 Nov 6;15(11):e0241932. doi: 10.1371/journal.pone.0241932 (PMC7647456; doi:10.1371/journal.pone.0241932)
Supplement: S1 Fig — (A) MA plot of DEGs. X-axis represents value A (log2 transformed mean expression level). Y-axis represents value M (log2 transformed fold change). Red dots represent up-regulated DEGs. Blue dots represent down-regulated DEGs. Gray points represent non-DEGs. (B) Pathway classification of DEGs. X axis represents number of DEG. Y axis represents functional classification of KEGG. There are seven branches for KEGG pathways: Cellular Processes, Environmental Information Processing, Genetic Information Processing, Human Disease, Metabolism, Organismal Systems and Drug Development (www.kegg.jp/kegg/kegg1.html). (PPTX) [file pone.0241932.s001.pptx]

## Slide 1
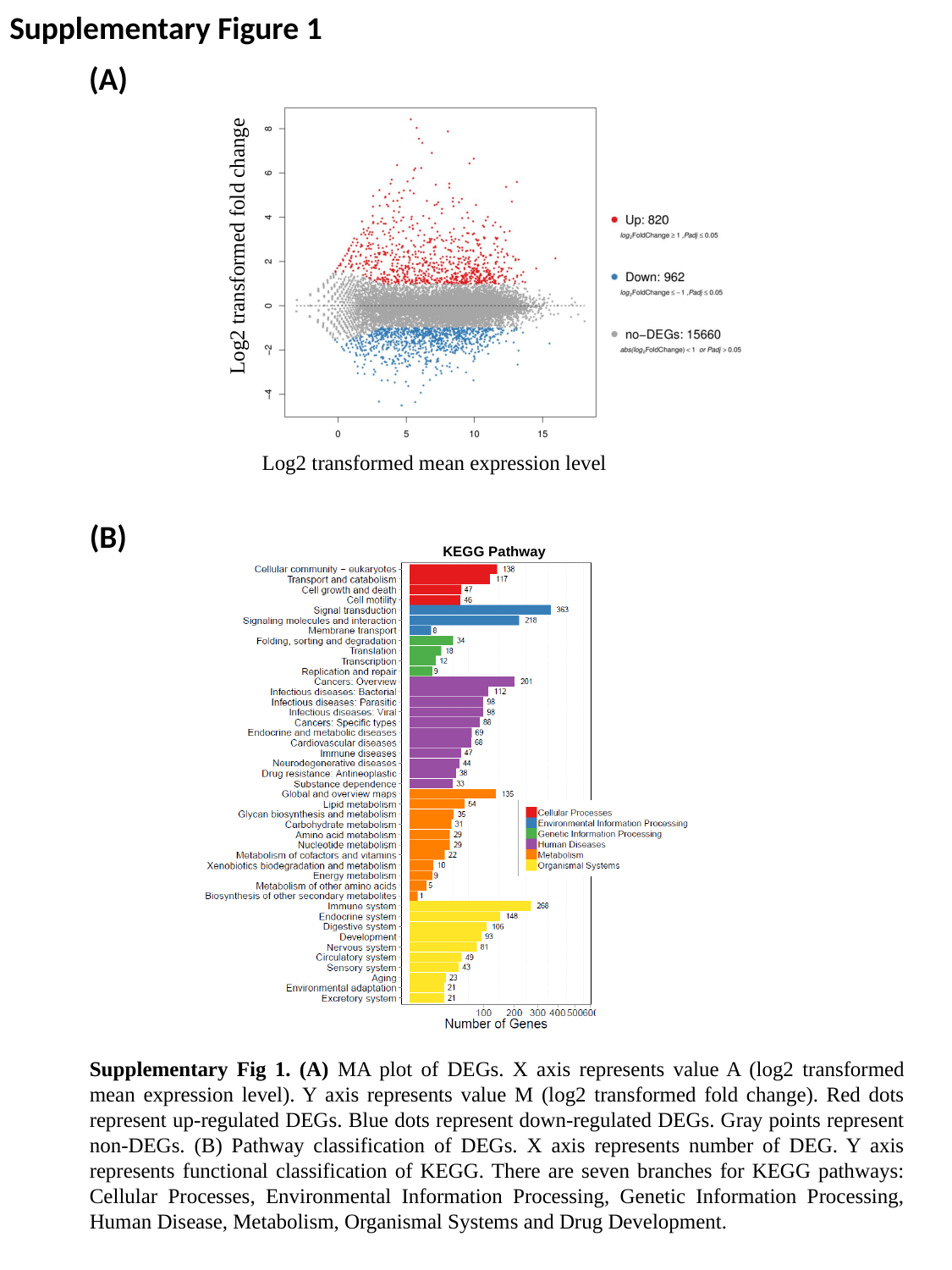

Supplementary Figure 1
(A)
Log2 transformed fold change
Log2 transformed mean expression level
(B)
KEGG Pathway
Supplementary Fig 1. (A) MA plot of DEGs. X axis represents value A (log2 transformed mean expression level). Y axis represents value M (log2 transformed fold change). Red dots represent up-regulated DEGs. Blue dots represent down-regulated DEGs. Gray points represent non-DEGs. (B) Pathway classification of DEGs. X axis represents number of DEG. Y axis represents functional classification of KEGG. There are seven branches for KEGG pathways: Cellular Processes, Environmental Information Processing, Genetic Information Processing, Human Disease, Metabolism, Organismal Systems and Drug Development.
