# Supplementary material for: Effects of stanniocalcin-1 overexpressing hepatocellular carcinoma cells on macrophage migration
Source: PLoS One. 2020 Nov 6;15(11):e0241932. doi: 10.1371/journal.pone.0241932 (PMC7647456; doi:10.1371/journal.pone.0241932)
Supplement: S2 Fig — PMA (5 nM) treated THP1 cells was seeded in cell culture inserts of 8 μm, and co-cultured with MHCC97L (with 200 ng/mL chemoattractant MCP-1) for 24 hr. Migrated THP-1 cells were stained in 0.5% crystal violet and countered using light microscopy. THP-1 cells treated with the inhibitor Y27632 (10 μM) and co-cultured with MHCC97L/P, showed a significant reduction in the migration (the left panel) as compared with the cells without the inhibitor treatment (the middle panel). The right panel showed the anti-migratory effects of MHCC97L/S1 on THP-1 cells. *P < 0.05 as compared with the respective control. (PPTX) [file pone.0241932.s002.pptx]

## Slide 1
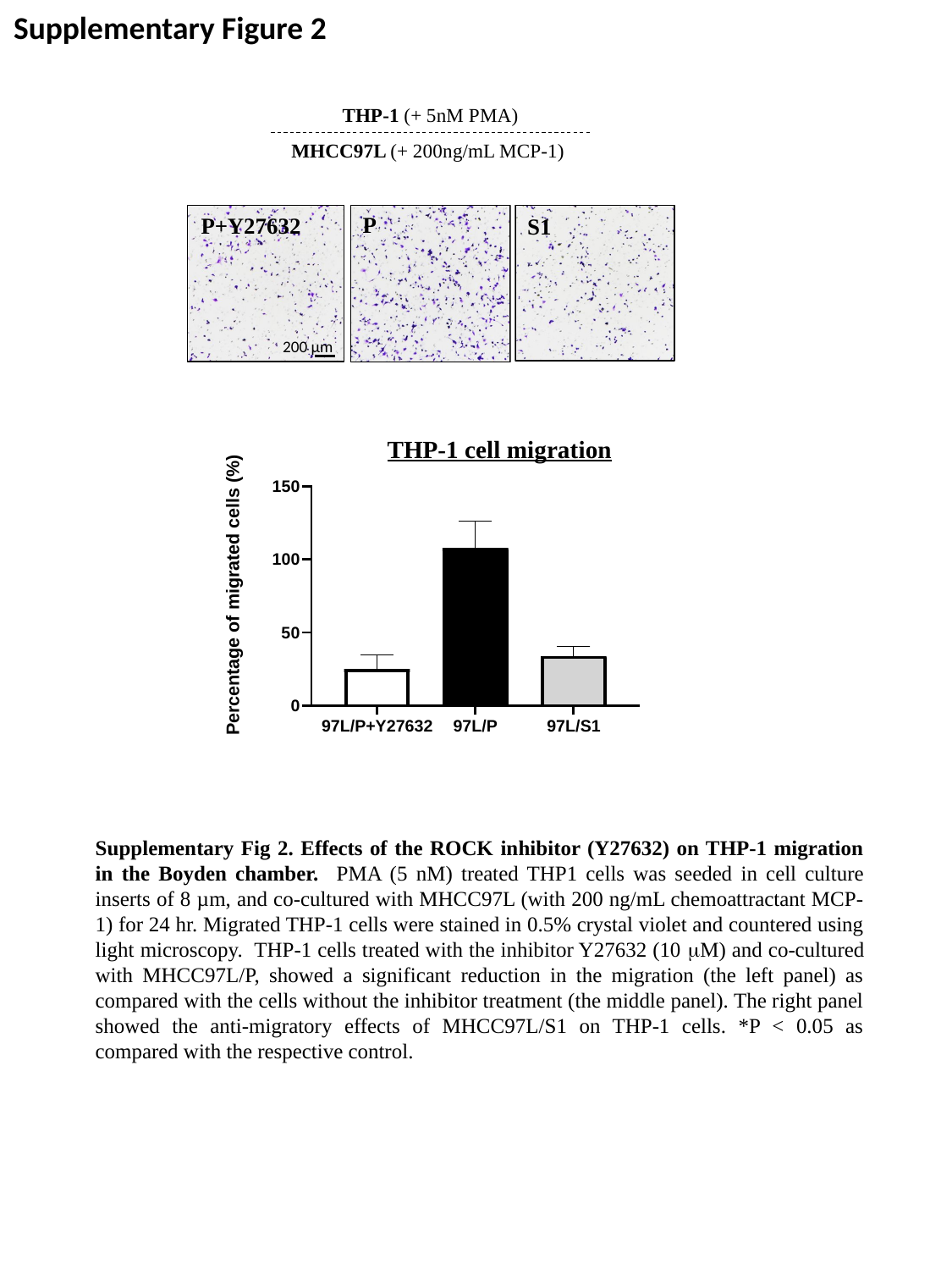

Supplementary Figure 2
THP-1 (+ 5nM PMA)
MHCC97L (+ 200ng/mL MCP-1)
P+Y27632
200 µm
P
S1
THP-1 cell migration
Supplementary Fig 2. Effects of the ROCK inhibitor (Y27632) on THP-1 migration in the Boyden chamber. PMA (5 nM) treated THP1 cells was seeded in cell culture inserts of 8 µm, and co-cultured with MHCC97L (with 200 ng/mL chemoattractant MCP-1) for 24 hr. Migrated THP-1 cells were stained in 0.5% crystal violet and countered using light microscopy. THP-1 cells treated with the inhibitor Y27632 (10 μM) and co-cultured with MHCC97L/P, showed a significant reduction in the migration (the left panel) as compared with the cells without the inhibitor treatment (the middle panel). The right panel showed the anti-migratory effects of MHCC97L/S1 on THP-1 cells. *P < 0.05 as compared with the respective control.
