# Supplementary material for: Effects of stanniocalcin-1 overexpressing hepatocellular carcinoma cells on macrophage migration
Source: PLoS One. 2020 Nov 6;15(11):e0241932. doi: 10.1371/journal.pone.0241932 (PMC7647456; doi:10.1371/journal.pone.0241932)
Supplement: S1 Raw images — (PPTX) [file pone.0241932.s006.pptx]

## Slide 1
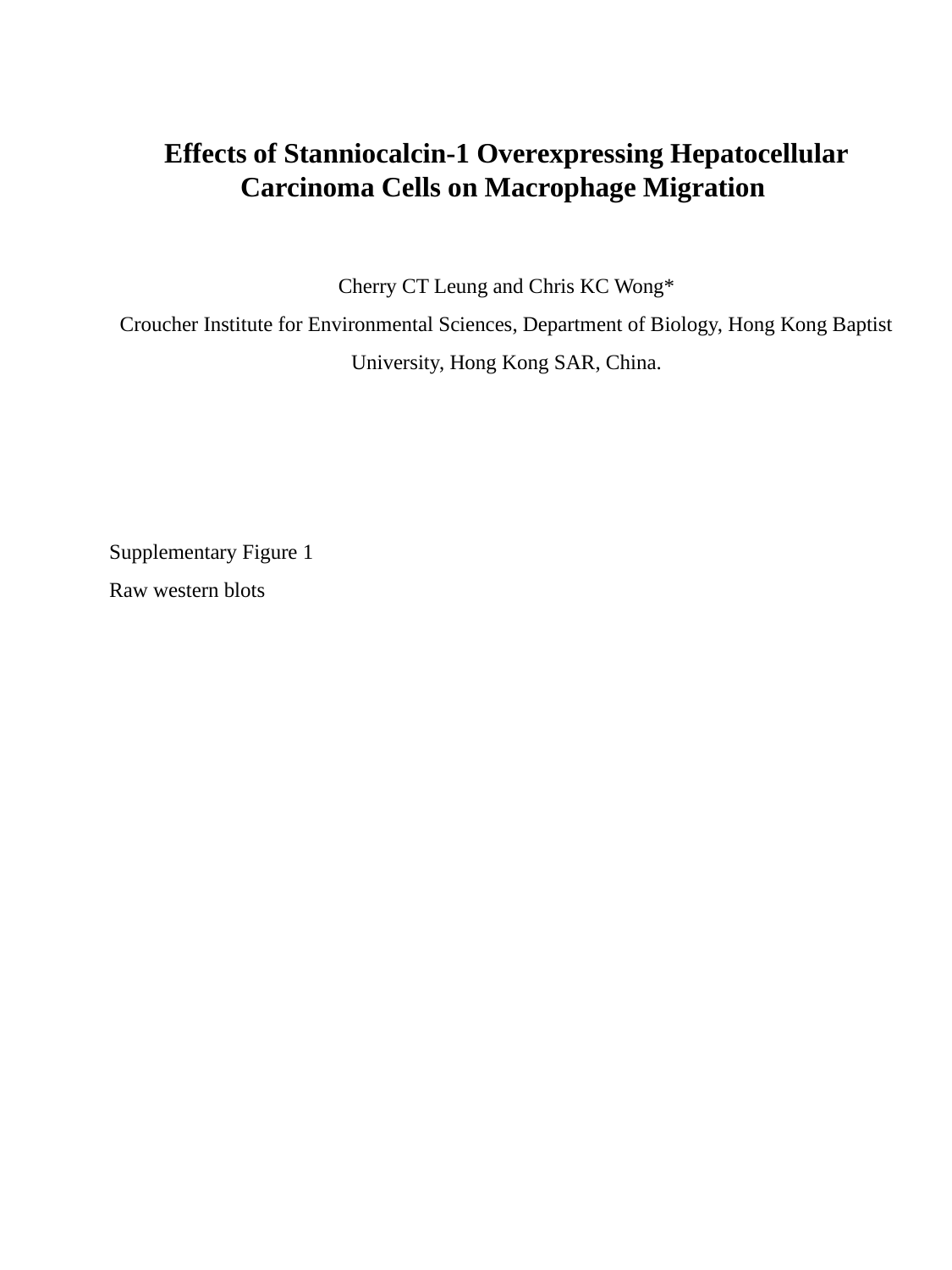

Effects of Stanniocalcin-1 Overexpressing Hepatocellular Carcinoma Cells on Macrophage Migration
Cherry CT Leung and Chris KC Wong*
Croucher Institute for Environmental Sciences, Department of Biology, Hong Kong Baptist University, Hong Kong SAR, China.
Supplementary Figure 1
Raw western blots

## Slide 2
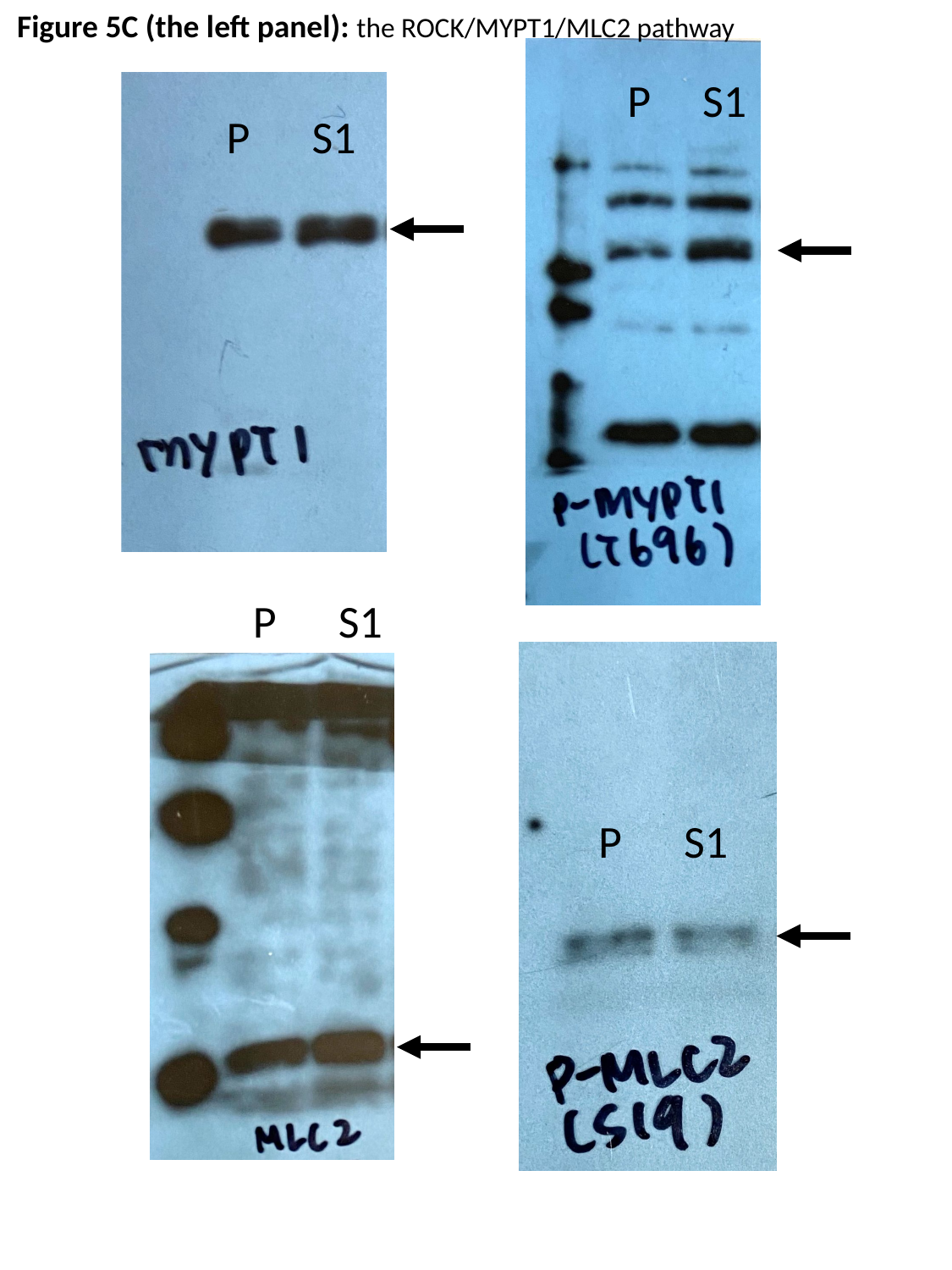

Figure 5C (the left panel): the ROCK/MYPT1/MLC2 pathway
P S1
P S1
P S1
P S1

## Slide 3
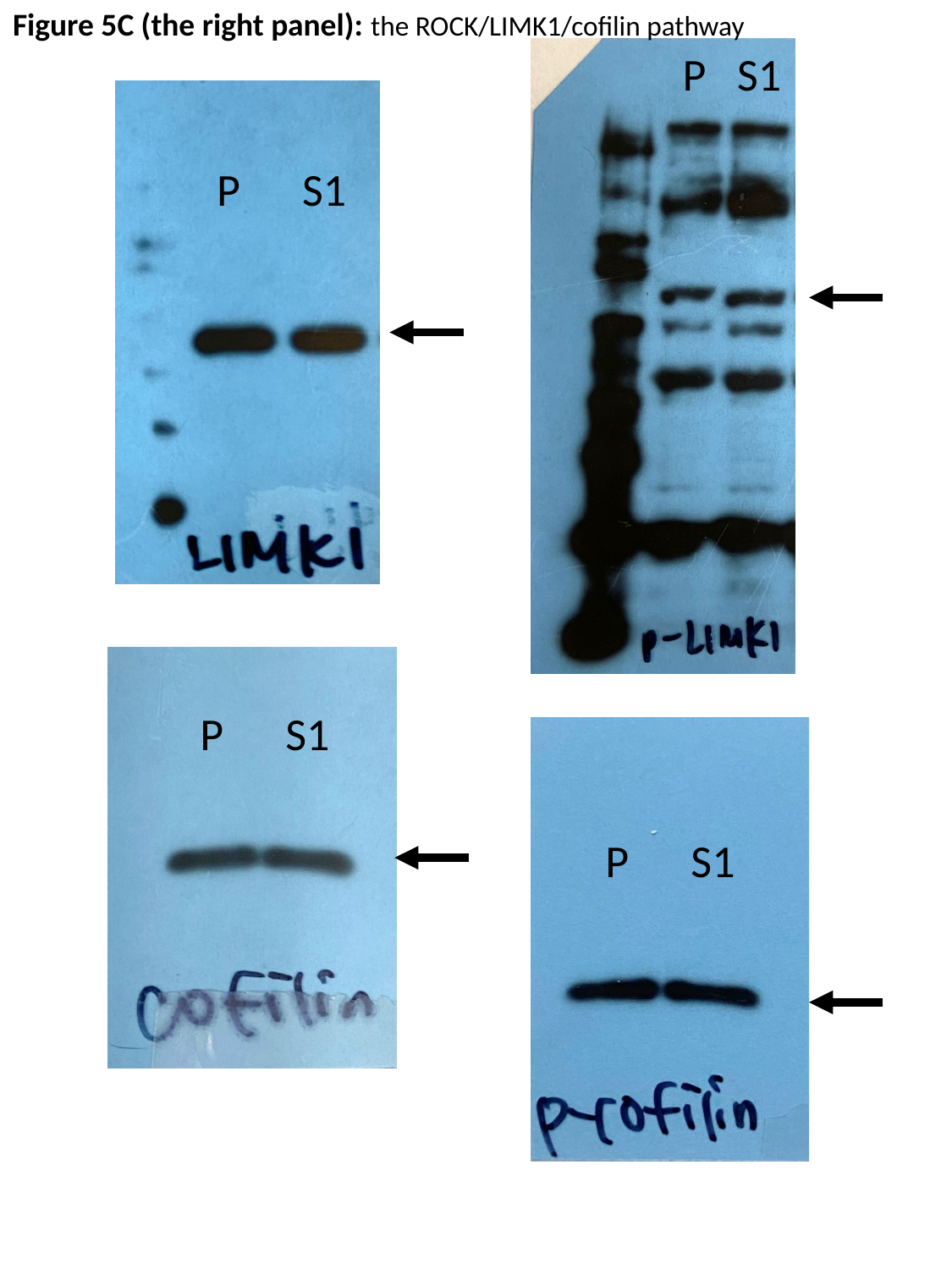

Figure 5C (the right panel): the ROCK/LIMK1/cofilin pathway
P S1
P S1
P S1
P S1

## Slide 4
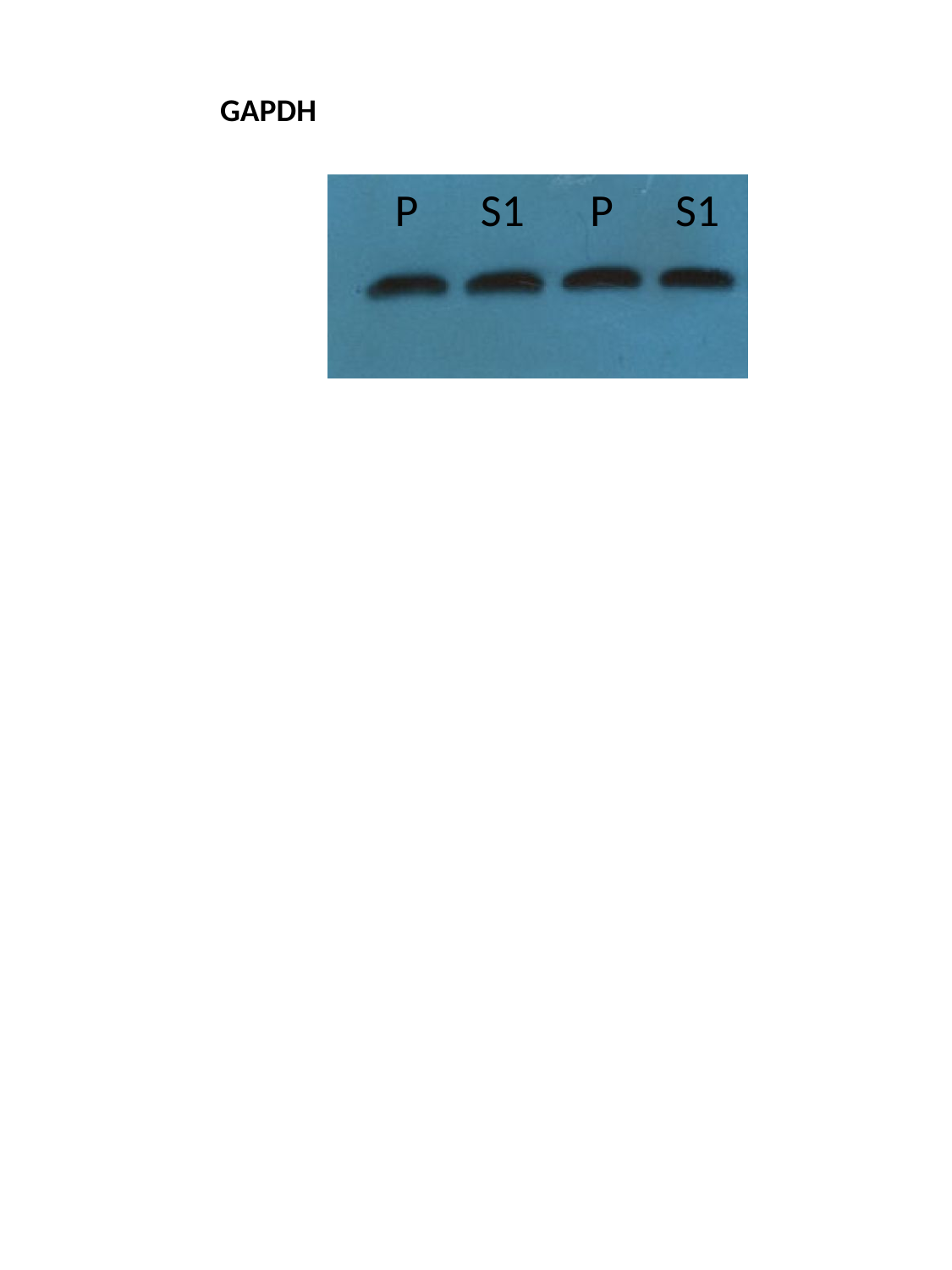

GAPDH
P S1
P S1
